# Iron deficiency and anemia in adolescent girls consuming predominantly plant-based diets in rural Ethiopia

**DOI:** 10.1038/s41598-019-53836-5

**Published:** 2019-11-21

**Authors:** Yohannes Seyoum, Christèle Humblot, Gaël Nicolas, Muriel Thomas, Kaleab Baye

**Affiliations:** 1 Center for Food Science and Nutrition, College of Natural and Computational Sciences, Addis Ababa University, Addis Ababa, PO Box 1176 Ethiopia; 2 IRD, UMR 204 Food and Nutrition Research in the Global South (NUTRIPASS), IRD/University of Montpellier/SupAgro, Montpellier, France; 3 INSERM UMR1149, CNRS ERL 8252, Centre de Recherche sur l’inflammation, Université Paris Diderot, Site Bichat, Sorbonne Paris Cité, 75018 Paris France; 4 Assistance Publique-Hôpitaux de Paris, Centre Français des Porphyries, Hôpital Louis Mourier, 92701 Colombes, Cedex France; 5 Laboratory of Excellence, GR-Ex, 75015 Paris, France; 6 Micalis Institute, INRA, AgroParisTech, Université Paris-Saclay, 78350 Jouy-en-Josas, France

**Keywords:** Nutrition, Risk factors

## Abstract

Rapid physical growth and the onset of menstruation during adolescence can increase the risk of iron deficiency (ID) and related adverse effects. However, little is known about the risk of anemia and ID among adolescent girls in Ethiopia. Therefore, we aimed to determine the prevalence of ID, low iron stores, and anemia and characterize selected risk factors in Huruta, Arsi Zone, Oromia Region, Ethiopia. A cross-sectional study was conducted among non-pregnant adolescent girls (15–19 years of age; n = 257). Data on household socio-demographic characteristics, anthropometric measurements, and women’s dietary diversity score (WDDS) were collected. Hemoglobin (Hb) and serum ferritin (SF), C-reactive protein (CRP), and α−1-acid-glycoprotein (AGP) concentrations were measured. Diets were predominantly plant-based, with a low consumption of animal source foods, fruits, and dark-green leafy vegetables. Only 4% of the adolescent girls had adequate dietary diversity (WDDS ≥5), and 35% were underweight. The prevalence of anemia (Hb <11 g/dL, 8.7%) and clinical ID (SF <15 µg/L, 8.7%) was low, but 41% had marginal iron stores (SF <50 µg/L). The low prevalence of ID, despite a predominantly plant-based diet is atypical and calls for adapted strategies to address low iron stores in this and other similar settings of Ethiopia.

## Introduction

Anemia affects a staggering two billion people worldwide^1^. Women of reproductive age in low- and middle-income countries (LMIC) are disproportionately affected. The etiology of anemia is multi-faceted and includes micronutrient deficiencies, chronic infections, and inherited hemoglobin disorders^2^. It has long been assumed that up to 50% of the anemia burden is related to iron deficiency (ID). However, recent studies have shown causal factors to be highly heterogeneous and suggested that the burden of anemia related to ID may be lower than previously assumed^3^.

ID is associated with impaired physical work capacity, cognitive function, and reproductive physiology and poor pregnancy outcomes^4^. To date, most initiatives that prevent or correct iron deficiency/anemia have focused on the first 1,000 days of life from conception to the second birthday of the child. However, growing evidence suggests that maternal nutrition before conception is as equally important for preventing adverse pregnancy outcomes^5^. For example, pre-conception iron-deficiency anemia (IDA) is associated with reduced infant growth and an increased risk of adverse pregnancy outcomes^6^.

The rapid growth that occurs during adolescence, the onset of menstruation, and the consumption of predominantly plant-based diets with low bioavailable iron, all contribute to the depletion of iron stores that substantially increase the susceptibility of adolescents to IDA and related adverse outcomes in future pregnancies^7^. Consequently, WHO is promoting weekly iron-folic acid supplementation programs in populations in which the prevalence of anemia in non-pregnant women of reproductive age is ≥20%^8^. Although there are increasing number of programs introducing intermittent (weekly) iron-folic acid supplementation for adolescent girls, there is little information on the prevalence of anemia, iron deficiency, and low iron stores for this population in many LMIC, including Ethiopia. Such information is critical for the design and implementation of effective intervention that maximizes benefits while minimizing possible adverse effects.

We aimed to determine the prevalence of anemia, iron deficiency, and low iron stores in adolescent girls (15 to 19 years old), using a cross-sectional design, in the Arsi Zone, Oromia Region, Ethiopia, where diets are predominantly plant-based.

## Results

### Characteristics of the study population

Among the 257 adolescent girls (age 15 to 19 years), 78% were enrolled in primary school (Table 1). The average household size was six and they were mostly male-headed (76%). Nearly all households had access to public tap water (96%) and 89% had a toilet facility (pit latrine with slab) on their premises. Half of the households had access to electricity. Wood was the primary source of energy for cooking (84%). Approximately, 83% of the households owned agricultural land and 71% livestock. Accordingly, agricultural production was the main source of income for their families.

**Table 1:** Household and respondents’ characteristics.

	Proportion(%) or mean ± SD
Household size (mean ± SD)	6.0 ± 2.0
Male-headed households	75.9
Household head education	
No formal education	23.4
Primary	48.0
Secondary	22.2
University or college diploma and degree	6.4
Household income source	
Wage income	5.9
Pension	1.2
Salary	10.2
Business	9.0
Agricultural production	73.7
Source of drinking water	
Improved source	95.4
Non-improved source	4.6
Type of toilet facility	
Improved facility	88.7
Non-improved facility	11.3
Access to electricity	50.0
Source of cooking fuel^§^	
Electricity	17.6
Charcoal	78.0
Wood	84.7
Straw/Shrubs/Grass	3.5
Animal dung	3.9
Fruits and vegetable cultivation	49.8
Use of cultivated fruits and vegetables (out of 49.8%)	
For sale	20.3
For food use	79.7
Ownership of agricultural land	83.1
Ownership of livestock	71.5
Mean age of participants (years ± SD)	16 ± 1.1
Respondent’s education	
Primary	77.6
Secondary	22.4

SD, standard deviation; ^§^multiple source of cooking fuel can be used by households; hence, options are not mutually exclusive.

### Anthropometric characteristics and food consumption

Approximately a third of the adolescent girls (35%) were underweight (BMI <18.5; Table 2). Only 4% consumed at least five food groups, which is considered to be the minimum cut-off for the WDDS. The mean number of food groups consumed was three.

**Table 2:** Anthropometry, hemoglobin level, and women’s dietary diversity score (WDDS) of adolescent girls.

	Proportion (%) or mean ± SD
Weight (kg)	47.6 ± 6.6
Height (cm)	155.4 ± 6.0
Body mass index (BMI)	
Underweight (<18.5)	34.6%
Overweight (25–29.99)	3.5%
WDDS	3.2 ± 0.6
WDDS category	
Adequate (≥5)	4.3%
Inadequate (<5)	95.7%
Hemoglobin (g/dL)	14.2 (1.0)

SD, standard deviation.

**Figure 1:**
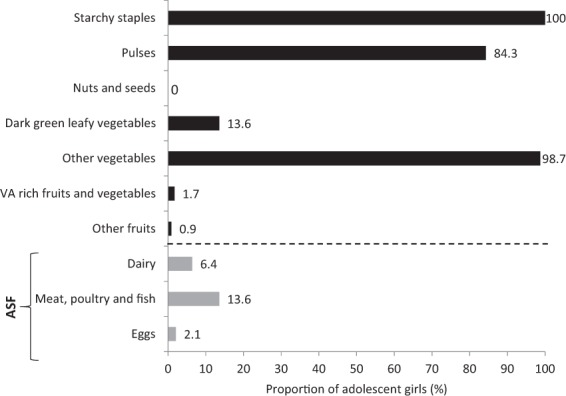
Food groups consumed according to the women’s dietary diversity score (WDDS) classification (n = 235). VA, vitamin A; ASF, animal-source foods.

Further investigation into the food groups showed that the diet of the girls was mainly composed of grains and tubers (100%), other vegetables (mainly onions; 99%), and pulses (84%; Fig. 1). The proportion of adolescents that consumed nuts and seeds, vitamin A-rich fruits and vegetables, dark green leafy vegetables, animal-source foods, and dairy products was very low.

### Anemia, infection and iron deficiency

**Figure 2:**
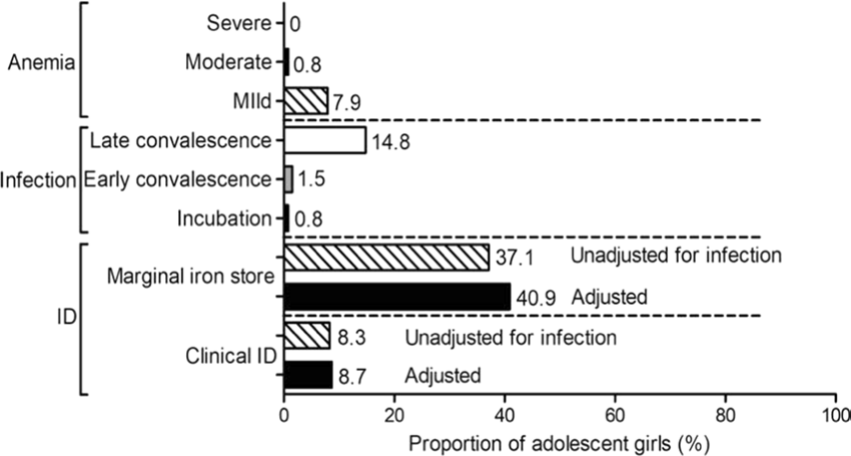
Prevalence of anemia, infection, and iron deficiency (adjusted/unadjusted for the presence/duration of infection/inflammation) in adolescent girls. ID, iron deficiency.

Despite a significant proportion of the study population being underweight and the low consumption of animal-source foods (ASF), the prevalence of anemia (8.7%) was very low, with no severe anemia, but 0.8% moderate and 7.9% mild anemia (Fig. 2).

The prevalence of clinical iron deficiency after adjusting for inflammation/infection based on serum CRP and AGP levels was low (8.7%; SF <15 μg/L), but 41% had marginal iron stores(SF <50 μg/L). The prevalence of infection in the area was 17%, of which 0.8%, 14.8%, and 1.5% were in incubation, early-convalescence, and late-convalescence phase, respectively. All of the anemic subjects were also iron deficient. Furthermore, correction of the ferritin values for inflammation did not significantly affect the estimate of the prevalence of iron deficiency (+0.4%) in this study.

## Discussion

We investigated the dietary diversity, prevalence of iron deficiency, and frequency of low iron stores of adolescent girls in rural Ethiopia. Diets were predominantly plant-based with little or no consumption of ASFs, fruits, or dark-green leafy vegetables. A considerable proportion of the adolescent girls were underweight, but surprisingly the prevalence of anemia and iron deficiency was low, with no cases of IDA or severe anemia.

The dietary diversity score was very low and was characterized by a very low proportion of adolescent girls consuming ASFs, fruits, and vegetables. This is in accordance with findings from the Ethiopian National Food Consumption Survey^9^. Such predominantly plant-based diets are poor sources of bioavailable iron, partially because of the high content of iron-absorption inhibitors, such as phytate and polyphenols^10,11^. Such diets, when compounded with the low consumption of fruit, which contributes to the intake of iron absorption enhancers, such as ascorbic acid, can increase the risk of IDA^10^.

Surprisingly, the prevalence of anemia and iron deficiency in this study was, based on the WHO classification, found to be a mild public health concern (8%) and was even lower than the national average (20%) for adolescents of 15 to 19 years of age^12^. This confirms the need for sub-national estimates and geospatial characterizations of anemia and its etiology. The relatively low prevalence of ID and anemia is in line with findings among more vulnerable age groups like young children and pregnant women in Ethiopia^13,14^. This is attributed to high iron intake, partially from soil contamination of major food grains^9,15^. Such soil contamination, attributed to the traditional threshing of grain under the hooves of cattle, was recently reported to contribute to the hemoglobin regeneration of iron-depleted rats^16^. However, there is limited knowledge on the bioavailability of iron from soil contamination and its interactions with the absorption of other nutrients and this subject warrants further investigation in humans^17^.

Although we observed no clinical iron deficiency, approximately half of the adolescent girls had marginal iron stores. As adolescent girls are future mothers and approximately 21 million adolescent girls in developing countries are expected to become pregnant every year, appropriate intervention strategies that restore iron stores are needed^18^. This is even more important in settings, such as rural Ethiopia, where pregnant women attend antenatal care late and are thus not able to start iron-folic acid supplementation sufficiently early^19^. Furthermore, a significant proportion of adolescent girls were underweight, suggesting that wider nutritional intervention that promotes dietary diversity and appropriate energy-balance is needed to reduce the risk of preterm birth and low birth weight^20^.

This study has a number of limitations that needs to be accounted when interpreting our findings: first, the cross-sectional design does not allow causal inference regarding the etiology of anemia and iron deficiency; second, although a battery of iron-related biomarkers were analysed, because of financial and technical constraints we were not able to analyze all nutrients whose deficiencies may be related to anemia. Ideally, analyses of both serum ferritin and sTfR would have provided the most effective assessment of the population’s iron status. This is because sTfR indicates the balance between cellular iron requirements and iron supply, while serum ferritin provides information on the iron stores. Unlike serum ferritin, sTfR is little affected by infection and inflammation and may also indicate the severity of iron insufficiency when iron stores are depleted and no other cause of erythropoiesis exists. However, given the low prevalence of depleted iron stores (SF <15 µg/L) and the correction of SF values for inflammation and infection by measuring both AGP and CRP, our assessment of iron status based on SF alone can still be considered accurate.

Notwithstanding the above limitations, this study contributes to the limited body of evidence on the prevalence of iron deficiency and low iron stores in adolescent girls in rural Ethiopia. In light of this finding, the most appropriate regimen of iron-folic acid supplementation that maximizes benefit while preventing adverse effects warrants investigation. Improving dietary quality through the promotion of diversified diet is not only critical for promoting healthy dietary patterns in the long-term, but also can support adolescents to enter pregnancy with adequate iron stores to support fetal development and their wellbeing.

## Methods

### Study area

The study was conducted in Huruta district, in the Oromia Region of Southeast Ethiopia, situated at 80^0^09′ north and 39^0^21′ east with an average elevation of ~1,978 m^21^. The study site was purposively selected for the baseline of a larger study that aimed to understand the etiology of anemia. According to the population census report of the CSA^21^, it has a total population of 15,298 of whom 7,463 are male and 7,835 female in its two *kebeles* (lowest administrative unit). Wheat is the major crop produced in the area. Cereals (barley, teff, and maize), legumes (horse beans and field peas), and various types of oil seeds are also cultivated. Furthermore, livestock, such as cattle, sheep, goats, and poultry are commonly raised in the area^22^.

### Study design and sampling

A cross-sectional study was conducted among adolescent girls (n = 257) aged 15 to 19 years. The sample size was calculated using a single-proportion formula for a finite population using OpenEpi (https://www.openepi.com/SampleSize/SSPropor.htm). A population size (total number of females) of 7,835, 20% prevalence of anemia for the Oromia Region^23^, 95% confidence level, and a design effect of 1.0 (because of random sampling) were taken into consideration for sample size calculation. The required sample size was augmented to account for 8% non-response rate. All participants were apparently healthy, with no chronic diseases, were not pregnant, and did not have any children. A listing of all the eligible adolescent girls was conducted with the help of the health extension workers. The study participants were then selected randomly from the listing of the two *kebeles* of Hururta.

### Ethical considerations

Ethical approval was obtained from the Ethics Review Committee of the College of Natural and Computational Sciences of Addis Ababa University (CNSDO/100/09/2016) and the Oromia Regional Health Bureau (AHIQFTFWO/408). Informed consent was obtained after a detailed explanation was given about the objectives and principles of the study. Informed consent/ascent was obtained from parents and/or legal guardians for those under 18 years of age and from adolescents only when they were above 18. All experiments were performed in accordance with relevant guidelines and regulations.

### Socio-demographic and anthropometric measurements

Data on socio-demographic characteristics was collected through interviews administered using a pre-tested questionnaire that included questions about the educational status of the family, household composition, available facilities in the household, and access to healthcare. The weight and height of each respondent were measured at the beginning of the survey using calibrated equipment, with the subjects wearing light clothing and no shoes. Then, the body mass index (BMI) was calculated as the weight in kilograms divided by the square of the height in meters (kg/m^2^). Accordingly, BMI values were categorized as underweight (<18.5), normal (18.5–24.99), and overweight (>25)^24^.

### Women’s dietary diversity score (WDDS)

The WDDS was estimated using open-ended qualitative 24-hour recall. All foods eaten, inside and/or outside the home, were recorded with frequent probing. A detailed list of all ingredients of the dishes, snacks, or other foods consumed was generated to enable the classification of mixed dishes. The foods consumed were then categorized into the ten food groups that constitute the women’s dietary diversity score^25^: (1) grains, white roots and tubers, and plantains (starchy staples); (2) pulses (beans, peas, and lentils); (3) nuts and seeds; (4) vitamin A-rich fruits and vegetables; (5) dark green leafy vegetables; (6) other vegetables; (7) other fruits; (8) meat, poultry, and fish; (9) dairy; and (10) eggs. Finally, the WDDS (0–10) was calculated by summing the number of food groups consumed in the previous 24 h^26^.

### Blood sample collection and hemoglobin determination

Non-fasting venous blood samples (~4 mL) were collected in BD Vacutainer®, Serum Separation Tubes (SST™) by experienced phlebotomists. A few drops of blood were immediately taken to measure hemoglobin (Hb) using the HemoCue® Hb 201^+^ system^27^. Then, the remaining sample was immediately centrifuged and aliquoted into 2-mL Eppendorf Tubes® that were stored at −20 °C until further analysis.

### Serum ferritin (SF), C-reactive protein (CRP), and α-1 acid-glycoprotein (AGP) analyses

SF was analyzed using a fully automated clinical analyzer electrochemiluminescence immunoassay (ECLIA, Elecsys® 2010 analyzer Cobas e 411; Roche Diagnostics GmbH, Mannheim, Germany). CRP and AGP were determined by immunoturbidimetric methods with a clinical chemistry analyzer (Cobas Integra 400 system; Roche Diagnostic GmbH) at the Ethiopian Public Health Institute. The coefficient of variation (inter-/intra-assay) was: hemoglobin (2.1%/ 4.5%), serum ferritin (3.1%/ 3.9%), CRP (1.2%/ 2.4%) and AGP (0.8%/ 1.6%).

### Deficiency cut-offs

The Hb values were adjusted for altitude and anemia was classified as mild (11–11.9 g/dL), moderate (8–10.9 g/dL), or severe (<8 g/dL)^28^. The presence and type of inflammation were determined using AGP and CRP values. Inflammation was considered to be at the incubation stage for CRP >5 mg/L and AGP ≤1 g/L, early convalescence for CRP >5 mg/L and AGP >1 g/L, and late convalescence for CRP ≤5 mg/L and AGP >1 g/L^29^. SF was corrected for inflammation by multiplying by 0.77 (incubation), 0.53 (early convalescence), or 0.75 (late convalescence). ID was defined as SF <15 µg/L and marginal iron stores as SF <50 µg/L. IDA was defined as a combination of anemia and ID^30^.

### Statistical analyses

Statistical analyses of the data were performed using SPSS for windows (IBM SPSS, Inc version 20). Descriptive statistics, such as the frequency (%), means, and standard deviations, are presented. The normal distribution of data was assessed using the Shapiro-Wilk test. P-values < 0.05 were considered statistically significant.
